# Appearance of Experience as Form and Process

**DOI:** 10.1007/s12124-020-09526-3

**Published:** 2020-04-25

**Authors:** Mårten Kae Paulsen

**Affiliations:** Department of Social Work and Guidance, Inland Norway University of Applied Sciences, Lillehammer, PO Box 400, N-2418 Elverum, Norway

**Keywords:** Recursion, Sensing, Perception, Reflection, Creativity

## Abstract

Theories of experience guide an understanding of how people’s conceptions of physical objects, events, and ideas are structured and organized. This article reviews how experience is understood as a phenomenon, a concept, and a category of phenomenology, pragmatics, and the experiential learning theory. Based on the review, the article discusses how existing theories of experience appear as static in a systemic view and are enriched when a dynamic view is added. The phenomenon of experience is analyzed as structured in recursive interactions between form and process in four layers, namely attention in sensing, categorization in perceiving, meaning in reflecting, and transformation in creating.

## Introduction

### Variation in Conceptions of Experience

Professionals in healthcare, social work, and education interact with people regarding their experiences. Their conceptions of people’s experiences influence their ability to perceive the needs of individuals seeking their help. Therefore, an essential task is to know the principles of how the conceptions of experience vary among professionals. Theories of experience guide an understanding of how people’s conceptions are structured and organized. However, this article differentiates between static and dynamic views of the conceptions of experience. It shows that existing theories appear as static in a systemic view in the sense that they do not clarify how interactions among experiences change over time. It claims that existing theories are enriched when researchers apply a dynamic view to capture change over time. This perspective provides new knowledge of experience as a phenomenon, a concept, and a category of the experiential learning theory.

When professionals know how the conceptions of experience vary, they may be able to compare their own conceptions with alternatives based on diverse ways to make distinctions among them. The knowledge is useful as a tool to enrich the professionals’ perspectives on how to interact with people’s regarding their experiences. By enriching their perspectives, the professionals can develop a deeper understanding of the principles at work when they interact with a client concerning his or her experience. An expected consequence is that many professionals will be able to counsel, guide, and supervise clients whom they otherwise would have struggled to help. This outcome is achieved since they will have access to more perspectives when reflecting on their interactions with their clients.

### Significance of People’s Interactions with their Experiences

When professionals interact with people during conversations, the people’s experiences are usually significant in terms of how these affect their lives. People often know an experience as a feeling and a way of perceiving or thinking related to an event, an issue, a lack of skill, an understanding, or the alternatives available for taking action. A common way for people to respond to others’ stories is to recall a similar story, which they then share with the tellers (Bateson 1980). The tellers and the listeners acknowledge the communality of the experience. The conversation often continues with stories about how people in similar situations understood and acted on the consequences of their initial actions. On many occasions, people resolve their issues when sharing stories with other laypeople whom they trust. When their struggle continues and they make an appointment with a professional, the professional has to offer something else than just sharing experiences with the clients. Some principles must be at work in the conversation between the professional and the clients to make a difference in the way that the clients interact with their experiences. The principles may appear when the professional is conscious of his or her conceptions of the clients’ experiences and how he or she uses these conceptions to influence and guide his or her practice.

### The Study

The purpose of this study is to explore and understand experience as a phenomenon, a concept, and a category of the experiential learning theory. The study aims to construct a model of the dynamics of how people recognize and interact with experiences, which can be their own or those of others. The model describes a structure of how people process their experiencing. To construct a model, the constituents of an experience need to be known. This study looks for these constituents in the processes of the interactions between the individual and the environment and how these processes are perceived in a person’s consciousness. This article describes the processes from the perspectives of the systems theory, cognitive linguistics, and phenomenology.

The study aims to contribute to the knowledge of how professionals structure and organize their conceptions of people’s experiences when they listen to the people’s stories. The stories mediate the interpersonal and the intrapersonal processes that interact when people recall their experiences. Professionals can help people structure and organize the conceptions of their stories in a variety of ways, depending on the professionals’ personal experiences, knowledge, beliefs, habits, and preferences.

This study’s expected outcome is to offer an understanding of the structure of experiencing, which professionals can use to compare and reflect on their own conceptions of people’s experiences. The model makes possible new distinctions among established conceptions of how people undergo their experiences. The model’s usefulness is that it may increase the number of alternatives accessible to professionals when systematically interacting with their clients concerning their experiences.

This article does not argue for a single true and right model of how to conceive of the clients’ experiences. Rather, it recognizes a diversity of ways to consciously represent an experience. The model’s advantage is its simplicity, as well as its ability to capture the structure of experience as both process and form..

### A Review of Theories of Experience

The general understanding is that experiences are manifested in intrapersonal and interpersonal interactions embedded in an environment. Intrapersonal interactions occur in people’s bodies and minds. Interpersonal interactions occur among persons in relationships with others, which are embedded in social, natural, cultural, and pragmatic environments. Experiences are recognized in human consciousness as conceptions of objects and events in the real or the imaginary world. With this general understanding as the background, this article reviews how experience is understood as a phenomenon, a concept, and a category in pragmatics, phenomenology, and the experiential learning theory. Pragmatics and phenomenology deeply influence how experience is understood in experiential learning. Experiential learning theorists have expanded their understanding based on pragmatics and phenomenology and have developed new distinctions to capture the relevant phenomena in the interface between experience and learning (Jarvis 2006; Kolb 2015; Mezirow 2009).

#### Pragmatic View

John Dewey (1938) exposes a pragmatic view of experience. Based on his understanding, the core conceptions are situation, environment, continuity, and time. Situations are characterized by interactions among an individual, objects, and other persons. The concept refers to people living in a world that they construct and organize in a series of situations (Dewey 1938, p. 42). Dewey (1938, p. 38) understands all human experiences as ultimately social. In these social relationships, people live and acknowledge themselves and the worlds to which they belong (Elkjær 2009, p. 78). For Dewey, experience is primarily an active–passive affair, not mainly cognitive.

The interactions comprise ongoing transactions between individuals and their environment, where any normal experience involves an interplay between internal and external conditions (Dewey 1938, p. 42). Therefore, the experience is not limited to a process unfolding within a person (Dewey 1938, p. 39). Sources outside an individual also give rise to the experience.

Dewey (1938, p. 35) links a principle of continuity to experience, where every experience both takes up something from the past and modifies in some way the imagination of future generations. What people experience in the present moment has a personal history as its knowledge base and context. When people experience their possibilities for the future, such experiencing interacts with similar experiences in the past, as well as the lack of them. Dewey’s view is that people discriminate among and sort experiences in situations; at the same time, the situations are connected and nested in a continuum of time. He uses the concept of organic circle to refer to the activity where people interact with one another and support, change, and create their social relationships and culture. Organic refers to subjects as active parts of their social and natural worlds. Based on his understanding of organic circles, Dewey argues against analyzing people’s actions as mechanical in the sequence of sensory stimulus, idea, and action. His view is that the interaction between the subject and his or her world should be considered.

Dewey’s concept of experience involves time as a dimension, which includes history and future possibilities. He pays attention to how various experiences interact over time, across the past, the present, and the future. Every experience influences to some degree the objective conditions under which further experiences are conceived (Dewey 1938, p. 37).

#### Phenomenological View

In the phenomenological view, experience differs from a biological and a psychological understanding (Gallagher and Zahavi 2007; Marton and Booth 1997; Thompson 2007). A biological understanding of experience refers to objective conditions for the experience as it can be explained by the exchange of matter, energy, and information in neurological networks. A psychological understanding of experience pertains to affective and cognitive functions, including memory, imagination, language, reason, and beliefs. The phenomenological understanding of experience refers to how objects appear in people’s sensing, perception, and meaning making. According to Lakoff and Johnson (1999, pp. 508–509), the difference between the phenomenological and the biological/psychological understanding involves four characteristic aspects. The first aspect is *color*. People experience an object’s color even though color is not a real part of the object but a result of human neurology. The second aspect is *space*, which people experience as structured. People can see borders between a surface and its regions, paths, center and periphery, foreground and background, and so on. However, space has no such structures. The structures are created in people’s experiences by the image’s schematic structures in human neurology. The third aspect is *time*, which people experience in movements, in increasing and decreasing resources, and as seasons, encompassing the past, the present, and the future. None of these qualities is an essential part of time. The phenomena emerge from people’s subjective experiences. The fourth aspect is *balance*. People can experience an imbalance of power in social relationships and an injustice in judgments between right and wrong. They can also feel forced to act against their will. None of these experiences is an essential dimension of an objective reality. The experiences have their origin in people’s embodied actions (Thompson 2007).

#### Experience in Learning Theory

Peter Jarvis makes a prominent contribution to understanding experience in the learning theory. In “Human learning,” he asks, “What is the nature of experience?” (Jarvis 2006, p. 71). He answers the question with four categories in a basic view and three more in an expanded view. The first category is *consciousness*, which enables people to be in the world and to know it. People are aware of their experiences through their sensations, feelings, and thoughts. Citing Chalmers (1996, p. 11), Jarvis understands consciousness from two perspectives of the mind—the psychological and the phenomenological. The psychological understanding perceives the concept of the mind as a causal or an explanatory basis for behavior. The behavior is described and interpreted from an observer’s position. The event or the situation is perceived from a bird’s eye view. In the phenomenological understanding, the concept of the mind is perceived as a conscious experience and a mental state that is consciously experienced. The experiencer is in the flow of experiencing. An example of the difference between the psychological and the phenomenological understanding of experience is that the former refers to an activity where the experiencer stops and reflects on something within the stream of experience, whereas the latter involves the experiencer in the flow of the present moment.

The second category in Jarvis’ (2006, p. 73) understanding of experience is *biography*. Biography refers to a person’s life history, where the person has the possibility to acknowledge an earlier experience’s or a potential future experience’s influence on a present experience. A biographical experience is the outcome of a person’s lifetime, which means that an experience in the present moment is embedded in a network of experiences in the course of a person’s lifetime. Individuals become aware of their biographical experiences in associations with and recall of earlier experiences in their lifetime.

Jarvis (2006, p. 73) calls the third category *episodic*. An episodic experience characterizes a person’s contact and interaction with an object, a person, or an idea in the world. Such experiences involve a person’s encounters with events and situations in the present, which may occur through both physical and mental activities. Normally, the duration of an episodic experience ranges from mere seconds to days. It is an interaction that has a beginning, a thematic interlude, and an ending.

Jarvis (2006, p. 74) labels the fourth category *sensation*, which refers to people’s ability to sense the world (as their environment), other people, and their bodies. Hearing, seeing, smelling, tasting, feeling, and balancing receive information from people’s environment. Sensation involves an ongoing monitoring of people’s present physical, mental, and social states. It is directed toward people’s physical and social environments, that is, to an external world. However, people also have sensations of themselves, experienced in their emotional and mental states. The sensation is thus directed to an internal world. The sensing occurs in the interface between their bodies and the environment. Peoples’ sense organs make it possible for them to process information and adjust to events that is occurring in their environment.

The fifth category includes *awareness and disjuncture*. People experience being aware when they are attracted to a sensation of something in their natural and cultural environments. To live is to be in a natural and social environment where people live in the flow of time and take their world for granted. Regarding the concept of disjunction, Jarvis (2006, p. 77) refers to how people encounter an event where they do not immediately distinguish how to respond to or think about the situation. The experience is a perturbation in an ongoing sensing of the environment.

The sixth category comprises *interest and perception*. Interest refers to how a person is engaged with an object, an event, a situation, or an idea. Perception pertains to how a person observes, identifies, or recognizes the cited elements. Interest and perception interact by their mutual influence. People focus on the objects and the events in their environment that are significant to them. The way that such objects and events have significance for people influences how they perceive the actual phenomena (Noë 2005). Thus, researchers may ask, “What do people experience when they deal with their interest and perception?” It is the moment when they acknowledge what a phenomenon is. It occurs in the moment before each person decides on “what its meaning is for me.”

The seventh category of experience encompasses *interpretation and meaning*. Interpretation involves people’s reflection on what an object or a phenomenon may mean for them. The underlying question for each person is “how an event or an object concerns me.” The purpose of the interpretation is to develop the meaning of an object or an event in a situation where people act and interact.

#### Summary of the Theories of Experience

In the pragmatic view, experience has a correlation to the real world. Persons undergo experiences in their interactions with physical objects, living organisms, and ideas in their environment, as well as in their bodies, feelings, and thoughts. Experience is bound to time by connecting actual or potential events and situations in the past, the present, and the future. Experiences are continuous and united yet differentiated. Experience is subjective in the sense that each person undergoes it. The pragmatic view recognizes knowledge as a subset of experience.

The pragmatic view of experience is not controversial compared with phenomenology and Jarvis’ (2006) theory of experiential learning. The view of embodied actions in phenomenology shares an understanding of experience with the pragmatic view. A difference appears when phenomenology focus on the structures of the subjective experience as color, space, time, and balance. Jarvis has also built his experiential learning theory on ideas from pragmatism. Two of his categories of experience—biography and episodic—are similar to the ideas proposed in the pragmatic view. As mentioned, episodic refers to a person’s contact with an object, a person, or an idea in the world. This category is similar to the pragmatic view that experience is correlated to the real world. Biography refers to lifetime experiences of situations in the past, the present, and the future. This category is similar to the pragmatic idea that experience is time binding.

The phenomenological view of how the experience of a phenomenon appears as experience is understood by the conception of embodied actions (Thompson 2007). These actions refer to how experience is structured by how the mind perceives the qualities of color, space, time, and balance. Physical objects, living organisms, and ideas appear in people’s experiences, influenced by their intentions. People bring their experiences to their consciousness and thereby reflect on, interact with, make meaning of, understand, know, and act on these experiences. Jarvis’ (2006) understanding of experience comprises the following four categories that are relevant to knowing how a phenomenon appears as experience: sensation, awareness and disjuncture, interest and perception, and interpretation and meaning. A pragmatic view of how a phenomenon appears is not as explicit as a phenomenological view and as Jarvis’ theory.

### Lack of Distinction among Conceptions of Experience

The review of theories shows refined distinctions among conceptions and categorizations of experience as a phenomenon. A common feature of the distinctions is their reference to static aspects of the experience. There is a lack of reference to the dynamic aspects. If researchers consider both aspects, their conceptions of experience will be enriched. Such insight leads to an exploration of this question: “How does people’s experience of a phenomenon appear as experience?”

### The Thesis

#### Experience of the Phenomenon of Experiencing

When people relate to their awareness of subjective, objective, and normative phenomena, they vary in their recognition of the phenomenon as either a form or a process (Bateson 1980, p. 209). Form and process refer to two qualities of experiencing. When experienced as a form, a phenomenon is recognized as static, where the relations between parts and between parts and the whole do not change over time. When experienced as a process, a phenomenon is experienced as temporal, where the relations between parts and between parts and the whole change over time.

Form and process are engaged in recursive interactions (Bateson 1980; Harries-Jones 1995). Recursive refers to a process that repeats itself. The output of the process feeds back as an input to the same process. Therefore, the result of the process depends on the foregoing repetitions. The recursion between the form and the process implies a process where the experience of the form determines the experience of the process, and the experience of the process determines the experience of the form in a self-referencing process.

**Fig. 1:**
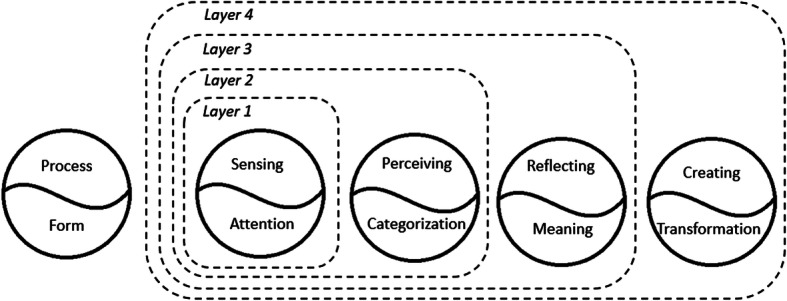
Layers of form (F) and process (P) in experiencing. Layer 1 comprise attention (F) and sensing (P). Layer 2 include layer 1 and add categorization (F) and perceiving (P). Layer 3 include layer 1–2 and add meaning (F) and reflecting (P). Layer 4 include layer 1–3 and add transformation (F) and creating (P)

The following sections explore how the recursion between form and process influences a person’s experience (See Fig. 1). Four layers and their interactions are differentiated, as follows: 1) attention in sensing, 2) categorization in perceiving, 3) meaning in reflecting, 4) transformation in creating, and 5) interactions among the layers.

The four layers appear in experience when people explore the structure of their experience in a metacognitive view. A common experience among people is to have sensations of the events in their environment, perceive what they sense, and use reason to decide how they will be affected by what they perceive. Sometimes, the meaning of an event is not immediately clear. People exist in a domain of the unknown and depend on creative thinking to transform the unknown into the known (Peterson 1999). Sensing, perceiving, reflecting, and creating refer to processes occurring in the mind. These processes are conceptualized as taking a static form when people connect sensing to attention, perceiving to categorization, reflecting to meaning making, and creating to transformation. The fifth domain involves an exploration of how experience appears when the first four layers interact.

#### The Thesis Statement

The thesis statement is that the phenomenon of experience appears as structured in recursive interactions between form and process in the layers of attention in sensing, categorization in perceiving, meaning in reflecting, and transformation in creating. This article discusses the thesis from the perspective of how an experience in each of the four layers appears to a person.

## Interaction Between Form and Process in Experience

### Attention in Sensing

In the recursive process between attention and sensing, people’s attention depends on what they are sensing, and what they are sensing depends on their attention.

#### Attention

Attention refers to an experience of a way of being in people’s interactions with their embodied selves and their imaginary and real worlds (Wu 2014, p. 110). Attention denotes an experience of people’s readiness to encounter incidents within themselves or in their environments. People feel their attention as the absence of the expected sensing or as a change in the structure or the intensity of the sensing. An example is the case of a parent and a child, where the child is left to play by herself or himself. From time to time, parents share stories about their experience of “suspicious silence,” which drew their attention when the background sounds were muted. Similarly, the parents became attentive to the sounds that broke through the background noise. It occurred when the parents or their children attracted each other’s attention with their voices. Their voices’ intensity, volume, and pitch passed through the background noise. The amounts of information that people sense go beyond what they can be conscious of (Nørretranders 1992). Attention *freezes* elements and sequences in a stream of sensory information. Attention prioritizes the appropriate area to focus on when sensing ongoing interactions with oneself or the environment.

#### Sensing

Sensing occurs in the interface between the human body and its environment. People receive sensory information in a continuous stream from the real world (physiosphere, biosphere, and noosphere). The senses respond to a limited aspect of the potential information as light, sound, energy, matter, and gravity. Sensing is experienced as sight, hearing, smell, taste, and kinesthetic or tactile feelings.

#### The Recursive Interaction Between Attention and Sensing

The recursion between attention and sensing is experienced in single sensory modalities or in combinations of them (visual, auditory, olfactory, gustatory, and somatosensory). An example is the case of a person attracted by a feeling. The feeling alone provides little information for the person to know what is going on. By shifting one’s attention to other sensory modalities, the person can supply the necessary amount of information and more precisely decide on what is occurring.

### Categorization in Perceiving

In the recursive process between categorization and perceiving the way people categorize objects and events depend on how they perceive them, and how they are perceiving them depends on how they categorize the objects and events.

#### Categorization

The criteria for membership in a category in basic-level theory for categorization are 1) the attributes that people attach to material objects or organisms, 2) shape what they perceive holistically, and 3) are the motor movements performed when they interact with material objects or organisms (Ungerer and Schmid 1996, pp. 66–69). Basic-level categorization refers to how people perceive the most obvious differences between material objects and organisms in their environment (Lakoff 1987).

People experience a category in immediate perceptions of objects in the real world and phenomena in the imaginary world. Not all phenomena that appear in experience refer to conceptions of concrete items. Examples of concepts that belong to abstract categories are morality, dignity, trust, love, caring, and learning. A relevant question is how the criteria (attributes, shape, and motor movement) apply when people categorize abstract phenomena. Lakoff (1987) shows that abstract categories are formed in two separate processes or in a combination of both processes.

In the first process, people project their experiences of a physical domain to an abstract domain. A physical domain consists of concrete objects. An abstract domain comprises ideas referring to phenomena as they are conceived in the real or the imaginary world. These perceptions have structures that correspond to attributes, shapes, or motor movements.

In the second process, a projection is performed from a basic-level category to a more general or a more differentiated category. When people project their experiences from a basic-level category to a more general level, they shift from using structures consisting of attributes, shapes, or motor movements at the basic level to structures comprising entities, functions, processes, levels, and relations at a more general level. Similarly, people project structures from a basic level to a more differentiated level.

Caring as a category can illustrate these processes. Peoples’ experience of the idea of caring has its origin in a physical domain, expressed in how people bodily experience other persons caring for them. Food, clothing, shelter, and warmth are examples of the attributes of the objects that characterize caring. Touch, light, temperature, and voice quality are examples of the shapes that characterize caring. Examples of motor movement include how hands touch when a body is cared for, tranquility, cooperation in the movement between a parent and a child, and rhythm in respiration. Examples of projections from a basic-level category to a more general category are projections of caring in personal relations to understand the qualities of caring in institutions or society as it occurs in kindergartens, schools, or nursing homes.

Another alternative is that new categories are formed by a projection from caring to a more differentiated category. It may occur in an examination of a person who needs care and is in an extraordinary life situation. The person’s needs include food, clothing, social integration, socialization, learning, or personal assistance.

#### Perceiving

The act of perceiving seeks patterns and structures in sensory-based information (Noë 2005). The sensory-based information can have its origin in the real or the imaginary world (Kosslyn et al. 2006; Thompson 2007). When people relate to others in the real world, the others are placed in the same physical space as concrete human beings. The perceiving of the others entails a mental process where the others are usually experienced in a three-dimensional space, in color/black/white/gray, in movements, in the discrimination between the foreground and the background, or as the relations between physical objects and organisms. When people relate to others in an imaginary world, they relate to the others in their memories or fantasies of past relations that could have been realized or future relations that could but do not need to be realized. In an imaginary world, the relations between physical objects and persons are principally organized in the same way as in the real world but with an essential difference. The difference is expressed in the degrees of freedom to enrich or reduce the way that people experience an experience in the imaginary world. In this case, there are no necessary bindings to factual events in the real world. The information that stimulates the senses relative to the imaginary world does not come from the external real world but from internal self-referential mental processes (Damasio 1999; Noë 2005; Thompson 2007).

People know the experience of perceiving as different from sensing in terms of the awareness of the alternation in the attention between the real and the imaginary world. The difference between sensing and perceiving is an abstraction. Both phenomena are sequences in a mental process that usually occurs faster than human comprehension. This situation makes it a challenge to discriminate between sensing and perceiving. People can discriminate during occasions when the mental process is halted. It occurs when people are aware of their feeling that they have forgotten something but at the same time cannot recognize what the item is. Before this feeling lets them know that something is missing, they sense that the feeling needs their attention. At the precise moment when they know that something is missing, they perceive. The process continues until they retrieve the item from the mind’s eye.

#### The Recursive Interaction Between Categorization and Perceiving

People experience the recursive interaction between categorization and perceiving in their selective conceptions of objects or events in the real world or phenomena in imaginary world. In their perceptions people construct representations of phenomena’s permanence and change in temporal processes (Noë 2005). People form these processes by their intentions to know what “it” is, where the “it” is a phenomenon, object or event. Peoples’ experience of how the “it” is formed (attribute, shape, and motor movement), and by that categorized, influences how it is perceived. The recursive process between categorization and perceiving continues until people decide on what “it” is.

An example of a recursive process is how peoples’ expectations of what to experience influences how they experience a certain object or event. People participating in social cooperation who are attentive to others’ mistakes will probably discover mistakes. Persons who are attentive to what others achieve in positive terms will probably discover what they achieve.

### Meaning in Reflecting

In the recursive process between meaning and reflecting the way people make meaning of objects, events, and ideas depends on how they reflect on them, and how they are reflecting on them depends on how they make meaning of the objects, events, and ideas.

#### Meaning

The concept of the meaning refers to an experience of a complex phenomenon that a person understands. Understanding does not differ from interpretation, explained by Gadamer as follows: “Interpretation is not an occasional, post facto supplement to understanding; rather, understanding is always interpretation, and hence interpretation is the explicit form of understanding” (Gadamer 2004, p. 306). He elaborates on understanding when he states, “Understanding is to be thought of less as a subjective act than as participating in an event of tradition, a process of transmission in which the past and present are constantly mediated” (Gadamer 2004, p. 291). Understanding a complex phenomenon implies taking part in a present experience and perceiving that experience from a historical perspective (Bruner 1990, p. 67). Such participation includes categorizing the phenomenon to identify the interactions among its categories, and in this way, interprets the phenomenon. Interpretations create a multitude of meanings of an incident, depending on the perspectives and the contexts in which the incident is regarded (Alvesson and Sköldberg 1994; Gadamer 2004; Nerheim 1996). The meaning of an interpretation is experienced by the person who interprets a particular phenomenon. Interpretations relate to what persons subjectively experience or to the valid intersubjective norms followed by a community’s members.

People experience meaning when whole/part aspects of a phenomenon are interacting in coherence (Bruner 1990, pp. 120–123). An example is a narrative as a unit of meaning. In its simple form, a narrative is a story with a beginning, an intermediate part where actions take place, and an end. In the narrative, the characters express their experiences as stories. The elements of the narrative include persons (or their substitutes), the context, the setting, actions, motives, intentions, hopes, relations, roles, contradictions, and emotions. In the narrative, the meaning of a phenomenon pertains to the whole aspect when the explanations of the phenomenon is consistently expressed in propositions. The part as an aspect of meaning refers to a situation where neither its explanations nor its interpretations are finite. They must be explored and developed to attain consistency and be free of contradictions.

#### Reflecting

Reflective thinking attend to people’s feelings and imaginative conceptions of their experiences. In a mode of reflecting people can enrich or delimit their consciousness of the experience and explore it by systematically vary how the experience is experienced. People become conscious of their experiences by feeling them, telling them in stories, and constructing imaginary symbolic representations of the event experienced. When people are reflecting on an experience they obtain insight by intuition and/or reason. The reflective thinking may be triggered by a feeling of a disturbance as lack of coherence in the experience of a complex phenomenon. By reflection on the experience people explore and discover what prevents the coherence among processes in a complex phenomenon. The intentions of reflective thinking are to construct meaning. The outcome of reflective thinking is an enriched and meaningful representation of the experience where people explore how others and own intentions could have been better cared for in alternative ways of communication or actions.

##### Intuition

Intuitive thinking brings to consciousness peoples’ conception of an experience and recalls relevant memories that people associate with the experience. The sources for intuitive thinking are peoples’ lived experience where experiences in the present are interacting with experiences in the past and possible experiences in the future. In the interactions the diverse experiences may inform each other and influence how people make meaning of an experience.

The theory of conceptual metaphor (Fauconnier and Turner 2002; Lakoff and Johnson 1999) explain how people use intuitive thinking to make meaning. Conceptual metaphors are applied in reflections when people try to understand or make meaning of phenomena with a high degree of complexity. Similarly, all individual experiences with others, situations, incidents, and the environment are the results of complex interactions between intrapersonal and interpersonal processes. To simplify the complexity of the multitude of everyday-life events, people use mental processes to create meaning in this multitude. Conceptual metaphors are employed in these mental processes.

People who experience the caring of another can reflect on the act by being aware that the other has “warm hands” and “friendly eyes,” experiencing “the feeling of a debt of gratitude”, or characterizing the other as an “angel.” The first conceptual metaphor expressed here refers to *warm hands*. People use their sensorimotor experience of temperature to conceptualize an experience of their relation to another person. In the second conceptual metaphor, *friendly eyes*, people use their sociometric experience of meeting the other’s gaze and eyes to characterize the relation as “friendly.” In the third conceptual metaphor, *the feeling of a debt of gratitude*, the same sensorimotor experience as people use in the economic domain (through an exchange of values) characterizes caring in the relation between the two persons. In the last example, the sensorimotor experience with an *angel* characterizes caring.

##### Reason

Reasoning is a way of reflecting on conceptions of an experience. When reasoning, people use language to guide their thinking (Mercier and Sperber 2017, p. 154). The purpose of logical reasoning is to argue for consistency among arguments that support or rejects claims (Toulmin 2003) and synthesizing and evaluating the meaning of the conception. The reflections may be guided by abduction, induction and deduction (Bateson 1980, p. 157). They may also be guided by critical thinking where people question the credibility of ideas and assumption. By reflection by reasoning people may discover and resolve contradictions among interpretations of their experiences.

#### The Recursive Interaction Between Meaning and Reflecting

People experience the recursion between meaning and reflecting in terms of how construction of meaning influences reflecting and how reflections influence construction of meaning. People’s consciousness of the recursion depends on whether and how they pay attention to the phenomenon of constructing meaning. The recursive process of constructing meaning proceed through reflecting by intuition and reason.

In their intuitive reflections people use metaphors to discover similarities and differences among experiences in an associative memory. In everyday life, they usually do not pay attention to the metaphors they use to create meaning (Lakoff and Johnson 2003). However, the construction of meaning is influenced. An example how the construction of meaning happen is in the metaphors of caring. When a person ascribes meaning to be cared for with the feeling of “a debt of gratitude” metaphor, the action is understood in the same structure as the exchange of values in an economic domain. The person who receives the care may feel obliged to somehow exchange a value for the action. When caring assigns meaning with “the warm hands” or “the friendly eyes” metaphor, the presence of another human comes to the foreground, with no hint of any obligation to exchange favors.

When people are reflecting by reason they use argumentation to strengthen or weakened their claims. The claims may refer to the intuitive reflections of the conceptions of an experience. The meanings of the experience emerge from asking why-questions where the answer often is an explanation of a complex phenomena. An example linked to the phenomenon of caring, is to ask: “Why some people associate caring to ‘warm hands’?” The question invite to further exploration of the meaning of caring.

The meaning of an experience is enriched by reflection. The meaning of the same experience often change depending on the context and frame the experience is considered from. Therefore the recursive interaction between reflecting and meaning can continue until a saturated meaning emerge. When a saturated meaning is constructed, people’s conception of the experience will not change with further variation of contexts and frames. The interpretation of an experience will not change despite variations of the contexts of the experience or in the positions and perspectives it is seen from. The interactions among recalled events in associative memory do not further enrich meaning.

### Transformation in Creating

In the recursive process between transformation and creating the way people transform their experience of objects, events, or ideas depend on how they are creating their conception of them, and how they are creating their conceptions depends on how they transforms their experience.

#### Transformation

People experience transformation when they change their identities, beliefs, attitudes, motivations, abilities to understand and act. In this change they become conscious of new ways of perceiving their environment, themselves and changes in how they process and create coherence in their experiencing. In this transformation, people create new knowledge of ideas, themselves, the objects and the social life in their environment. They increase their degrees of freedom to interpret and act. An example of the difference between established and new ways of understanding is the cognitive process of adaptation, where Piaget (1974) distinguishes between assimilation and accommodation. In an assimilative process, people understand themselves, objects and events in their environment, and ideas by means of established cognitive schemes (Piaget 1974, p. 10). When people undergo experiences that do not fit into established knowledge and understanding, an accommodative process is possible. New and more complex cognitive schemes arise in accommodation (Piaget 1974, p. 152).

#### Creating

In an experience of creating people attend to something in their life that it is important for them to understand, achieve or just create. People may be struggling with understanding and act on experienced contradictions in their life. When no meaning appears when people use habitual metaphors, categories, and attention in reflective thinking, the issue under question calls for a creative mind. A creative mind is also in play when people of pure curiosity and joy are exploring new ideas.

In the process of creating people are making unfamiliar combination of familiar ideas, or explore and transform conceptual spaces in their minds (Boden 2004, pp. 3–5). Conceptual spaces refer to structured styles of thought that people internalize from the culture. Fauconnier and Turner (2002) explain the process of creating using the concept of conceptual blending. Conceptual blending refer to the combination and recombination of experiences by conceptual metaphors. In conceptual blending, metaphors from two different domains of life experiences are blended in a mental process. The result of the process is a new third metaphor in which meaning is created for the focused experiences. In this mental process, the structures of the two blended metaphors interact and are transformed into a new structure that the person uses to create a new meaning of the focused experiences.

The metal process of creating occur in the interactions between a person’s thought and the sociocultural context (Csikszentmihalyi 1996, p. 23). A successful outcome of the process is a novel and original insight. The new insight is often experienced in a creative process as spontaneous, unpredictable and surprising.

#### The Recursive Interaction Between Transformation and Creating

People experience the recursion between transformation and creating in terms of how experience of transformation influences creating and how creating influence transformation. People’s consciousness of the recursion depends on whether they attend to their creativity and intend to play with possibilities, discover, or reconcile contradictions, or solve problems.

The experience of transformation interact with the process of creating. This happens when people expect and believe that transformation of an experience is possible. They have faith in themselves and their ability to change their situation by creating new insight. A faith that acknowledge the value of the transformation despite the fact that people know the present state without knowing possible future states. They do either not know what changes may come that result in a new way of processing experiences.

The process of creating interact with peoples’ transformation of their present experience. This happens in processes influenced by three different intentions. The first intention is creating by just have joy with playful exploring of new ideas and expressions. People are open to be surprised by what emerge from the activity. The second intention is to create coherence in part-whole organization among contradicting experiences to understand and act on an issue. People seek solutions or reconciliation among viewpoints on the issue. The third intention is to create solutions to problems connected to complex issues. People seek strategies to structure and process information that reveal differences that are essential to understand and act on the problem.

### Interactions among Several Ways of Experiencing

The mental processes of attention in sensing, categorization in perceiving, meaning in reflecting, and transformation in creating proceed in various durations, ranging from a fraction of a second to minutes, hours, days, and perhaps even years. Transformations in creating are experienced as surprising insights, often because of mental activities whose duration extends from hours to days, months, and years. When people encounter a phenomenon with curiosity, wonder, an inquiring attitude, or as attractive, it is implicit in the experience of the phenomenon that they pay attention to it, categorize it, and create and judge the meaning that it holds for them. They are usually unaware of all the sequences in the process of experiencing a phenomenon, especially when the phenomenon is well known to them. It is the opposite case when the phenomenon is new and hyper complex (as it usually is in scientific research and in conflicts among people and organizations), and people learn to master new skills or understand novel ideas. In such situations, people must be aware of how they pay attention to, categorize, or create meaning to continue their exploration and learning and do so successfully.

### How Does the Model of Interaction Between Form and Process Enrich Theories of Experience?

The recursive process *attention in sensing* bears similarities with Jarvis’ *consciousness*, *sensation*, and *awareness and disjuncture* categories. The experience of attention presupposes a conscious and focused sensing of an event, an issue, or an idea. Sensation may refer to an ongoing process of sensing or an awareness that attracts attention to some aspects of what is sensed. Part of a disjuncture is an experience of an interruption in a person’s expected flow of sensory information in a situation. A difference between *attention in sensing* and Jarvis’ categories is the former’s structure as a form and a process in recursive interactions.

An implication of professionals’ distinction between attention and sensing is an increase in their ability to help their clients to be aware of how they are sensing their experiences. With this awareness the clients, in collaboration with the professional, can explore if attention to other aspects of the experience make a difference that enrich the construction of the experience.

The recursive process *categorization in perceiving* has similarities with embodied action, knowledge as a subset of experience and consciousness, awareness and disjuncture, and interest and perception. Perception is embodied action (Noë 2005). Knowledge of what is perceived as physical objects, living systems, or ideas is based on complex systems of categorization. Categories emerge from how human communities agree on how to sense, perceive, and characterize a phenomenon. People’s awareness is evoked when they are exposed to phenomena that are new to them or when something occurs, and they do not immediately know what it is and how to respond to it. In a fraction of time, they must figure out what the phenomenon is and how to interact with it. In Jarvis’ theory, people’s varying degrees of interest influence how they perceive a phenomenon. This present article adds that people’s values likewise influence their perceptions. This kind of influence on perceiving appears in an alternative perspective that is discussed next in this article. The issue is relevant in a discussion of how interest and value interact with perception. The focus here is on the recursive interactions between categorization as a form and perceiving as a process, which can be discussed independently of the content of what is formed and processed.

*Categorization in perceiving* differs from the established theories of experience in the distinction between form and process and their recursive interactions. At the same time, the idea of embodied action, knowledge as a subset of experience, awareness and disjunction, and interest in perceiving are preserved in the new understanding. The categories are organized in a novel way.

An implication of professionals’ distinction between categorization and perceiving is an increase in their ability to help their clients to be conscious of how they categorize an experience and how they perceive the experience. A consciousness by the professionals and clients of how perceptions are processed and judged to belong to a category, open minds for exploration of other ways of perceiving and by that categorize an experience. In this exploration the experience is enriched with information.

The recursive process *meaning in reflecting* bears similarities with embodied action, the pragmatic view, and *interpretation and meaning*. People make meaning in a process, starting from what something is to how it concerns them. Meaning is felt (Gendlin 1997) in an embodied action (Thompson 2007; Varela et al. 1991). In the pragmatic view, people make meaning by telling others about their actual and imagined experiences from situations in the past, the present, and the future, as well as compare and interpret their experiences across time and situations. The means of comparing are metaphors, metonymies, and analogies. These means make it possible to explore how meaning can change when an experience is enriched by paying attention to different perspectives.

A difference between *meaning in reflecting* and the other perspectives is the former’s structure as a form and a process in recursive interactions. When people create their meanings they confirm their opinions of an event. They advance from a process of wondering and exploration to a firm opinion, which is a movement from a process to a form.

An implication of professionals’ distinction between meaning and reflection is an increase in their ability to help their clients to be conscious of the metaphors, analogies or reasons they use to experience meaning from their reflections. When clients are conscious of how they reflect to construct meaningful representations of their experiences, they at the same time have the possibility to explore how various ways of reflecting enrich the experience, create new meaning and understanding of an issue.

The recursive process *transformation in creating* shares similarities with *awareness and disjuncture*. People struggle to make meaning of some of their experiences. This situation occurs in what Jarvis calls disjuncture and is common in interpreting issues and creating knowledge. To resolve contradictions and their confusion, people usually compare an experience with earlier experiences in their life histories by seeking similarities. Sometimes, they lack a similar experience. They meet the challenge to transform their experience in creative ways.

A difference between *transformation in creating* and *disjuncture* is that the former creates the distinction between form and process. Disjuncture describes a form characterized by an experience where people don’t know the answer to a situation and what to do (Jarvis 2006, p. 78). The distinction makes it possible to pay attention to the recursive interactions between transformation and creativity. There is also a difference between the recursive view of experience and the pragmatic view that states, “Experience has a correlation to the world.” The recursive view of experience is extended to experiences that are not correlated to the real world. This is the case in creative interactions among imaginative conceptions in peoples’ minds. They can cognitively and emotionally experience conceptions of objects, events, and ideas that are not yet realized but can be realized in the future. The recursive process *transformation in creating* includes this kind of experience.

An implication of professionals’ distinction between transformation and creativity is an increase in their ability to help their clients to cope with bewilderment concerning contradictions in the clients’ experiences. The process of creating leads to transformation of bewilderment to clarity and new understanding. A challenge for the professionals are to cooperate with the client to establish a creative process that transforms clients experience of contradicting processes to a coherent understanding of the same processes. One mean to create coherent understanding is by negotiating among contradicting processes in the clients’ experience.

## Conclusion

This article’s contribution to new knowledge is the understanding of experience as form and process in the four layers, namely attention in sensing, categorization in perceiving, meaning in reflecting, and transformation in creation. Form and process are engaged in recursive interactions. The benefits of structuring experience in this way is that the structure is correlated with processes that unfold in real time when people access their experiences during conversations. By being aware of what people pay attention to in their conversations; how they categorize objects, events, and ideas; and make meaning of each experience, professionals can help them develop the experience. Such a process can occur by guiding people’s attention, ways of categorization, and uses of reflection and (when convenient) transforming the meaning of each experience by means of creativity.
